# Surgical experiences of final-year undergraduates and the impact on their career aspiration stratified by sex/gender

**DOI:** 10.3205/zma001683

**Published:** 2024-06-17

**Authors:** Angelika Homberg, Elisabeth Narciß, Udo Obertacke, Katrin Schüttpelz-Brauns

**Affiliations:** 1 Medical Faculty Mannheim, Heidelberg University, Division for Study and Teaching Development, Department of Medical Education Research, Mannheim, Germany; 2 Medical Faculty Mannheim, Heidelberg University, Division for Study and Teaching Development, Competence Center for final-year education, Mannheim, Germany; 3 University Medical Center Mannheim, Centre for Orthopaedics and Trauma Surgery, Mannheim, Germany

**Keywords:** career aspiration, gender differences, Germany, surgery, undergraduate education

## Abstract

**Objectives:**

Surgical undergraduate training takes place in a male-dominated work environment that struggles with recruitment problems. Experiences of cultural and sex/gender-specific barriers of women in surgery have been reported worldwide. Overall, the experiences that students have in coping with the emotional impact of surgery as a profession are thought to be crucial to their subsequent career choices. We investigated whether students' self-reported experiences differed by sex/gender in terms of frequency and content, and whether they were related to their career aspirations.

**Methods:**

In Germany, the final year represents the last part of the undergraduate medical study program. At the Mannheim Medical Faculty, a 12-week surgical training is mandatory. After completing their surgical training, the students were asked about their experiences and their later career aspirations. These experiences were analysed using quantitative content analysis. The relationship between the quality of experience and career aspirations as well as sex/gender differences were statistically measured.

**Results:**

In the 475 questionnaires analysed (response rate 52%), the number of positive and negative mentions does not differ by sex/gender. However, male students feel more actively involved and female students feel poorly briefed and think that supervisors are less interested in their training. A significant influence on career aspirations was found in the *performance* category for female and male students, and in the *supervision* category for female students.

**Conclusion:**

The positive experiences that students gain while performing surgical activities have an impact on their subsequent career choices. In particular, a good settling-in period and encouragement to actively participate could help to attract more women to surgery.

## Zusammenfassung

**Hintergrund:**

Die chirurgische Ausbildung findet in einem männlich dominierten Arbeitsumfeld statt, das mit Rekrutierungsproblemen konfrontiert ist. Weltweit wird über kulturelle und geschlechtsspezifische Barrieren für Frauen in der Chirurgie berichtet. Generell wird davon ausgegangen, dass die Erfahrungen, die Studierende bei der Bewältigung der emotionalen Anforderungen im Fachbereich Chirurgie machen, entscheidend für ihre spätere Berufswahl sind. Wir untersuchten, ob sich die selbstberichteten Erfahrungen der Studierenden in diesem Bereich in Bezug auf Häufigkeit und Inhalt geschlechtsspezifisch unterscheiden und ob ein Zusammenhang mit den späteren Berufswünschen besteht.

**Methoden:**

In Deutschland stellt das Praktische Jahr den letzten Abschnitt des Medizinstudiums dar. An der Medizinischen Fakultät Mannheim ist ein 12-wöchiger klinisch-praktischer Einsatz im Fachbereich Chirurgie obligatorisch. Die Studierenden wurden am Ende dieses Einsatzes über ihre Erlebnisse und ihre späteren Berufswünsche befragt. Die berichteten Erlebnisse wurden mittels quantitativer Inhaltsanalyse ausgewertet. Der Zusammenhang zwischen der Qualität der Erfahrungen und den Berufswünschen sowie die geschlechtsspezifischen Unterschiede wurden statistisch geprüft.

**Ergebnisse:**

475 Fragebögen wurden ausgewertet (Rücklaufquote 52%). Die Anzahl der positiven und negativen Nennungen unterscheidet sich zwischen den männlichen und weiblichen Studierenden nicht. Studenten berichten jedoch häufiger, aktiv eingebunden worden zu sein und Studentinnen fühlen sich weniger gut eingearbeitet und meinen eher, dass die Betreuungsperson nicht an ihrer Ausbildung interessiert ist. Ein signifikanter Einfluss auf die Berufswünsche wurde in der Kategorie *Praktisches Arbeiten* für weibliche und männliche und in der Kategorie *Supervision* nur für weibliche Studierende festgestellt.

**Schlussfolgerung:**

Die angenehmen Erfahrungen, die Studierende bei der Ausübung chirurgischer Tätigkeiten im Praktischen Jahr machen, wirken sich positiv auf ihre spätere Berufswahl in der Chirurgie aus. Insbesondere eine gute Einführungsphase und die Ermutigung zur aktiven Teilnahme könnten dazu beitragen, mehr Frauen für die Chirurgie zu gewinnen.

## 1. Introduction

The final year of medical school is an important time for the students, as medical knowledge is to be transformed into practice [1]. The field of surgery is firmly anchored in the training structure with a compulsory part in order to give all students, regardless of their later career choice, an insight into surgical practice. Learning in the operating room poses special challenges for students and teachers [2], as actions are strictly regulated by standardized surgical procedures, hygiene regulations and other measures to ensure patient safety. The particular challenge in surgery is to manage and cope with the emotional impact of surgery, the educational task, and the social relationships at work in the operating room [3]. Final-year students need appropriate support and supervision in order to make the transition from education to professional practice, avoid overload and thus increase patient safety [4]. Students’ experiences in meeting these challenges are expected to be critical to their subsequent career choices [5], [6], [7].

The shortage of surgical residents is expected to worsen in the coming years [8]. Although the number of women graduating from medical school exceeds the number of men in most countries, surgical specialities still fail to attract and retain women. This results in a loss of qualified female physicians that jeopardizes the maintenance of surgical care and reduces sex/gender diversity in surgical departments [9], [10]. 

Cultural and sex/gender-specific barriers to women in surgery have been reported worldwide [11], [12]. Hill et al. [13] claim that the character of surgery as a competitive, male-dominated speciality causes surgeons to appear intimidating and students, especially women, to lose interest in surgery as a result. Other factors influencing the decision to pursue a surgical career were the characteristics of surgical training and the student’s “fit” with the culture of surgery [14]. There is evidence that satisfaction with the integration into the team, the quality and structure of teaching, and the acquisition of competencies through final-year surgical training have a significant impact on the choice of the corresponding speciality [15]. A review published in 2014 shows that women are more likely to be discouraged from pursuing a surgical career if there is a lack of female role models, and that some women who aspired to a surgical career experienced sex/gender discrimination [16].

We assume that students’ experiences during their final year of study differ by sex/gender and influence their subsequent career choices. There are many more reviews that examine and list factors influencing career choices in surgery for women [17], [18], [19], but none that explicitly contrasts these factors with men. There is also a lack of studies that provide insight into the extent to which specific experiences influence career choices. 

We asked students after their final-year placement about positive and negative experiences during their surgical training and their later career aspirations and analysed the responses with regard to the following questions:

1. Do female and male students report negative and positive experiences with different frequency?
2. Do the reported experiences differ between female and male students with regard to the issues raised?
3. Does the quality of the reported experiences influence later career aspirations of female or male students?

The results can help uncover sex/gender disparities and reveal tangible ways to attract more students to choose surgery as their career path.

## 2. Materials and methods

### 2.1. Setting

At all German medical schools, students complete a mandatory surgical placement in their final year. This training begins after five years of undergraduate medical training and represents the last part of studies before the final examination. At the Medical Faculty Mannheim of Heidelberg University the final-year curriculum is divided into four parts of 12-week placements each during which students are required to take the subjects of surgery, internal medicine, ambulatory medicine, and one elective subject [20]. The order in which students take the subjects is assigned and students are placed in hospitals, at ambulatory clinics or at medical practices which are in close contact with the medical school and meet the same educational standards.

### 2.2. Data collection 

Since August 2011, all final-year students at the Medical Faculty Mannheim have been asked to participate in an online survey as part of the quality assurance process at the end of their placements. The questionnaire consists of approximately 80 questions on the following topics: demography (sex/gender, age), organization of final-year training, satisfaction with final-year deployment [21] and teaching, acquisition of medical skills. For this study, we analysed responses to the following two open-ended questions: “What were the most pleasant experiences at your most recent final-year placement?” and “What were your most unpleasant experiences at your most recent final-year placement?”

The responses on sex/gender (single-choice question, options: female, male, no response) were used to specify sex/gender differences. The option “diverse” was not offered during this survey period. Students’ sex/gender were evaluated to be able to compare the gender groups. Future career aspirations were recorded with a 5-point rating scale (statement: “I could imagine working in this speciality”; 1=I totally disagree, 5=I totally agree).

Each invitation email contained a link to the survey tool EvaSys and a personal transaction number (TAN). This procedure enabled an anonymous participation in the evaluation, whereby non-participation could be identified based on the unused TAN numbers. The final-year students were reminded twice.

We have strictly complied with the requirements of the Declaration of Helsinki (2013). The survey was conducted anonymously. Students were asked for their consent that the data collected could also be used for research purposes without any advantage or disadvantage to them as a result. This study included data from the cohorts that started their final year in the time from February 2013 to November 2020.

### 2.3. Data analysis

The answers to both open questions were analysed using Früh’s quantitative content analysis [22]. Following a previous study on final-year experiences [23], we deductively assigned all statements on the positive and negative experiences to the categories:

1. *performance and activities, *
2. *cooperation and team atmosphere, *
3. *supervision and guidance.*

In a second step, we counted the number of female and male students who had mentioned one or more aspects in these categories.

Sex/gender differences in the frequency of statements were calculated using chi-squared test for each of the two qualities in the individual categories. Here, the proportions of students who gave at least one answer each were compared. Quantitative analysis was performed with a Bonferroni-corrected p-value at the level of p<.008.

To further explore sex/gender-specific differences using quantitative content analysis [22], we inductively broke down the pleasant and unpleasant reports in each category into subcategories. We counted the respective number of mentions in each subcategory and analysed which aspects were prioritized among female and male students. 

The influence of the quality (pleasant or unpleasant) of experiences on career aspirations was measured using the Mann–Whitney U test for pairwise comparisons in each category for female and male students with a Bonferroni-corrected p-value at the level of p<.017. Responses from students who reported having experienced both qualities were not included in this test as they did not point in one direction.

## 3. Results

A total of 1,378 questionnaires were sent to medical students after their surgical placement, of which 721 were completed (response rate 52%). We excluded 128 questionnaires in which students had not consented to participate in the study, 110 that referred to a placement abroad due to different contextual conditions, and eight without information on their sex/gender. This resulted in a total of 475 questionnaires. Participating student characteristics are shown in table 1 (Tab. 1).

148 pleasant and 109 unpleasant statements were assigned to the category *performance and activities*, 109 pleasant and 106 unpleasant to *cooperation and team atmosphere*, and 89 pleasant and 71 unpleasant to *supervision and guidance*.

### 3.1. Frequency of reported experiences depending on sex/gender

175 female and 123 male students reported pleasant experiences, and 139 female and 107 male unpleasant ones. No differences were found when comparing the number of the responses in the categories for each quality using the chi-squared test. Results are shown in table 2 (Tab. 2).

### 3.2. Different issues raised in reported experiences

The analysis of the free-text responses showed that the 175 positive responses from women contained a total of 272 phrases (1.6 per response), and the 139 negative ones 210 phrases (1.5 per response). The 123 positive responses from men contained a total of 178 phrases (1.4 per response) and the 107 negative ones 129 phrases (1.2 per response). Based on the content analysis, five to nine subcategories were formed for each category. The subcategories and frequencies of mentions are shown in table 3 (Tab. 3), table 4 (Tab. 4) and table 5 (Tab. 5).

### 3.3. Influence of the quality of the experiences on career aspirations

**Figure 1:**
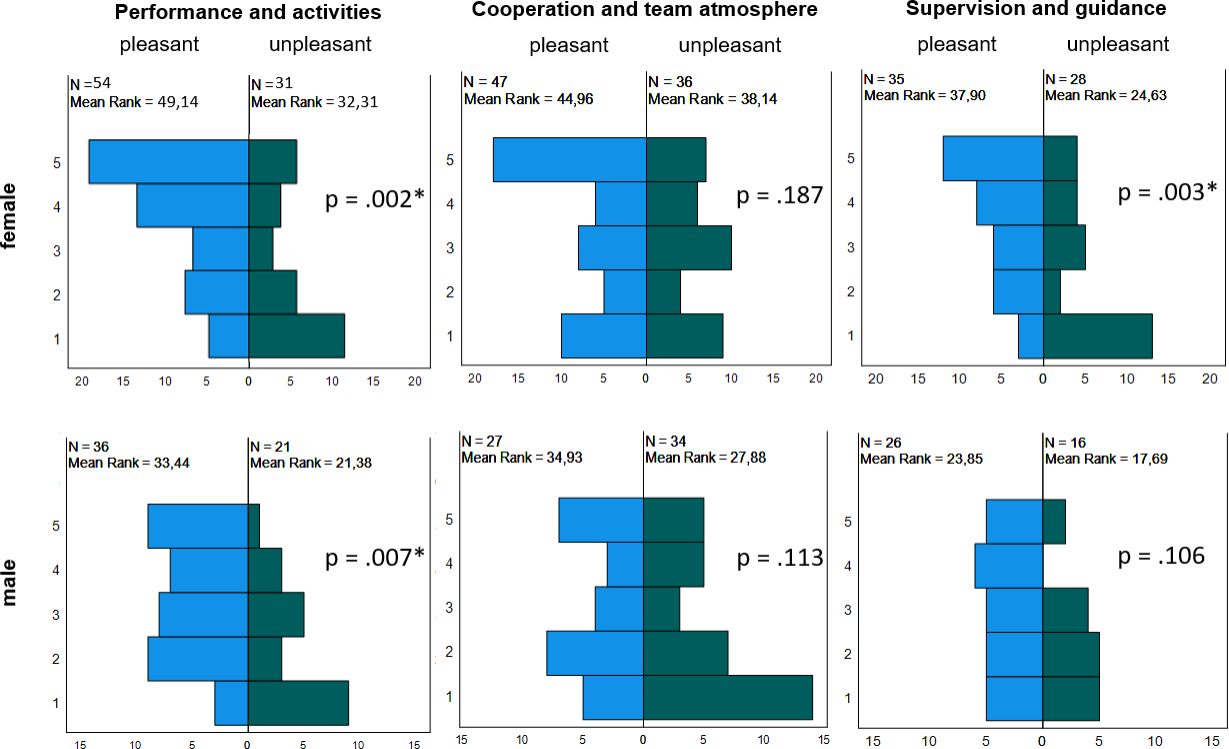
Influence of pleasant and unpleasant experiences of female and male final-year undergraduates on career aspiration *Note:* Mann-Whitney U test, *significant differences between career aspirations with p<.017 (after Bonferroni alpha adjustment). Rating question (y-axis): 1=I totally disagree, 5=I totally agree.

In order to calculate the influence of pleasant and unpleasant experiences on later career aspirations, questionnaires that addressed both qualities were excluded. These were n=57 in the *performance and activities *category, n=35 in the *cooperation and team atmosphere* category and n=27 in the *supervision and guidance* category. Significant results were found for male and female students in the category *performance and activities* and for female students in *supervision and guidance*. The results are shown in figure 1 (Fig. 1).

## 4. Discussion

Evaluating the experiences reported in 475 questionnaires made it possible to gain insight into sex/gender-specific issues and their influence on later career choices. The research questions are answered as follows:

### 4.1. Frequency of reported experiences depending on sex/gender

The number of positive and negative experiences among men and women do not differ in the individual areas. Most positive experiences were assigned to the *performance and activities* category. This shows that the students value the areas and activities where they can lend a hand and perform medical tasks themselves.

### 4.2. Different issues raised in reported experiences

The analysis of the subcategories also shows that men and women largely address the same subtopics. 

The further analysis of the category *performance and activities* shows that female and male students welcome the chance to perform hands-on activities, such as assisting and suturing and to work independently, but women more often report that they have nothing to do. Some women complain that they have not learned how to admit patients, while men do not address this topic. It is noticeable that men are more likely to report that they actively collaborate and get involved and that women are more likely to be in a situation where they can work independently. It is unclear whether women have fewer opportunities to participate actively or whether they hold back more because they have less confidence in their own capabilities.

In the area of *cooperation and team atmosphere*, female and male students emphasize a friendly team or nice doctors and both seem to suffer equally from an unfriendly atmosphere. A recent survey shows that interactions with students in surgery are sometimes rude or disrespectful [24]. However, inappropriate communication seems to be a problem in other medical specialities as well. In 2012, German medical students were surveyed online about esteem, verbally inappropriate treatment, and other forms of negative experiences. Of 391 students, 56% reported a lack of appreciation and 34% reported verbally inappropriate treatment. Most, but proportionately more women, felt that this was a burden [25].

In the area of *supervision and guidance*, it appears that women are more likely to be instructed and men are more likely to be supervised. While positive aspects were raised equally, almost all negative aspects were raised more frequently by female students, such as not being briefed or supervisors being disinterested in students’ training. There is slight evidence that women are more likely to feel poorly supervised than men. It is possible that the male-dominated environment still plays a role here and women are less likely to be perceived as future colleagues by supervisors or that they need more support to build self-confidence. It is also possible for cultural reasons that opposite sex/gender superiors are less likely to offer individual personal support [26].

### 4.3. Influence of the quality of the experiences on career aspirations

It was found that the experiences in *performing and activities* were directly related to later career choices in surgery for female and male students, and the experiences in *supervision and guidance* were related to later career choices for female students. The experiences gained from working as part of a team had no influence on subsequent career choices.

The immense influence of performance is also supported by O’Herrin’s finding that subsequent career choices in surgery may be correlated with the level of exposure to surgical procedures at the end of the study program [27]. Berman et al. showed that students who sutured during their assignment in the operating room were 4.8 times more likely to be interested in surgery and students who move the camera were 7.2 times more likely to be interested [28]. In retrospect, surveyed doctors of non-surgical specialities also named learning basic skills such as suturing and working under sterile conditions as the most valued learning content of non-graduate surgical training [29].

It was somewhat surprising that no influence on career aspirations could be shown for the *cooperation and team atmosphere* category. It is possible that in our study the effect was too weak to be proven. However, the frequency of mentions indicates that team atmosphere is nevertheless explicitly perceived and valued as a noteworthy experience. Even in reviews examining the determinants of career choices in surgery, no studies are found that identify team atmosphere as an influential factor [7], [17]. It is possible that students take into account that this factor depends more on the location than on the speciality itself. Quite a few final-year students rotate through different units during their surgical training. In some reports the positive or negative team experiences were explicitly related to only one of these units and may have been compensated by experiences in a different unit. However, integration into the team is cited as an important factor in assessing satisfaction with the surgical training in the final year [15].

Although the fewest experiences were attributed to the *supervision and guidance *category, an influence on their career choice could be derived for female students. It may be that female students are more dependent on good mentoring than male students because they have fewer role models, are thought to be less competent and therefore have fewer options to build self-confidence on their own [30], [31], [32]. Some faculties have good experiences with mentoring programs, as they have been shown to influence later career choices, especially for women [33], [34], [35].

### 4.4. Limitation 

This was a survey of final-year medical students in Germany. The experience in the last year of medical school probably influences their career choices more than in other countries where this decision is made earlier. Furthermore, our study was conducted at only one medical school. However, since sex/gender aspects in particular are highly dependent on culture, any transfer to other locations and countries is only possible to a limited extent. Differences and tendencies may also be influenced by generation rather than sex/gender [36].

A strength of our study is the large number of responses to open-ended questions, where respondents could give unbiased reports about their experiences. On the one hand, the amount of data allowed quantitative analysis and systematic comparisons; on the other hand, the informative value of free-text answers collected by questionnaires is limited [37]. It is possible that subtle differences are not captured in this study. Here, students reported prominent pleasant and unpleasant experiences only keyword-like. Less salient experiences or particularly sensitive aspects may not have been captured here because they were not reported or the students concerned did not participate in the survey. Furthermore, it is impossible to say whether the career aspirations of the individual students were already clear before the assignment or to what extent the corresponding speciality choice in turn influenced the experience. There are some studies that indicate that students decide on a speciality at an early stage of their training or even before medical school [36], [38]. However, it has also been shown that these decisions can change during their final year [5], [39]. 

We asked students about past experiences and calculated their influence on career aspirations. This enables the identification of career choice factors that students may be unaware of.

On the other hand, factors that are more focused on future working conditions are not revealed. There are studies, for example, showing that the opportunity to work academically is crucial for a career choice in surgery [40], [41] and that the decision to start a family has a significant influence against the choice [36]. In addition, there are numerous studies showing that the subsequent professional conditions in surgery are not advantageous, especially for pregnant women and mothers [42], [43]. This aspect was not considered as female students do not have any assignment in surgery during pregnancy and maternity leave. 

## 5. Conclusion

The results show that the students' experiences in their final year can have an impact on their later choice of surgery as a speciality, and therefore the recruitment of students should begin during their training. Final-year students welcome the opportunity to perform hands-on activities, such as assisting and suturing, and to work independently as part of a friendly team. Our study shows that the experiences students gain while performing surgical activities have an impact on their later career choices. For female students, subsequent career choices also depend on the perceived quality of supervision. In particular, a good settling-in period and encouragement to actively participate could help to attract more women to surgery. 

## Notes

### Funding

The research project was supported by the Federal Ministry of Education and Research, project number 01PL17011C. 

### Authors’ ORCIDs

- Angelika Homberg: [0000-0001-5585-1126]
- Katrin Schüttpelz-Brauns: [0000-0001-9004-0724]

## Acknowledgements

We are thankful to all the students who participated in this survey.

## Competing interests

The authors declare that they have no competing interests.

## Figures and Tables

**Table 1:**
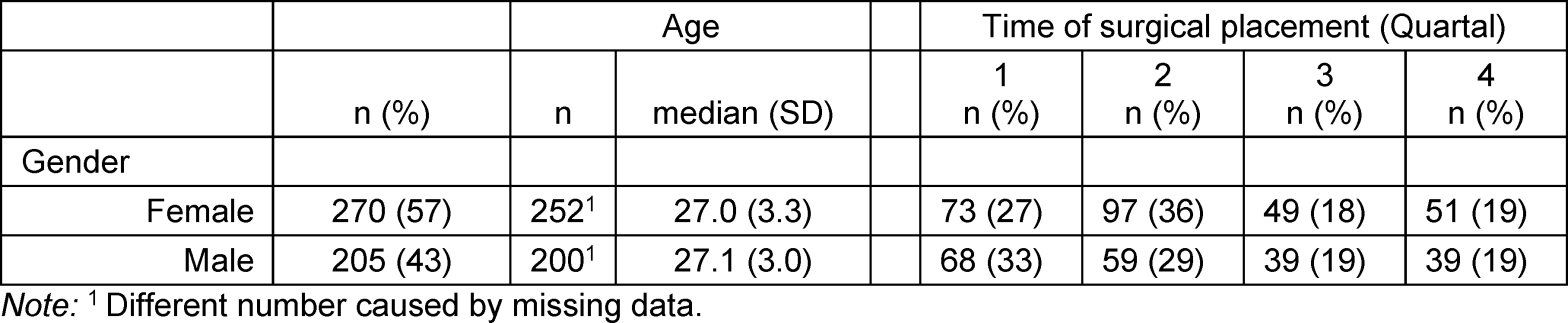
Student characteristics and time of surgical placement

**Table 2:**
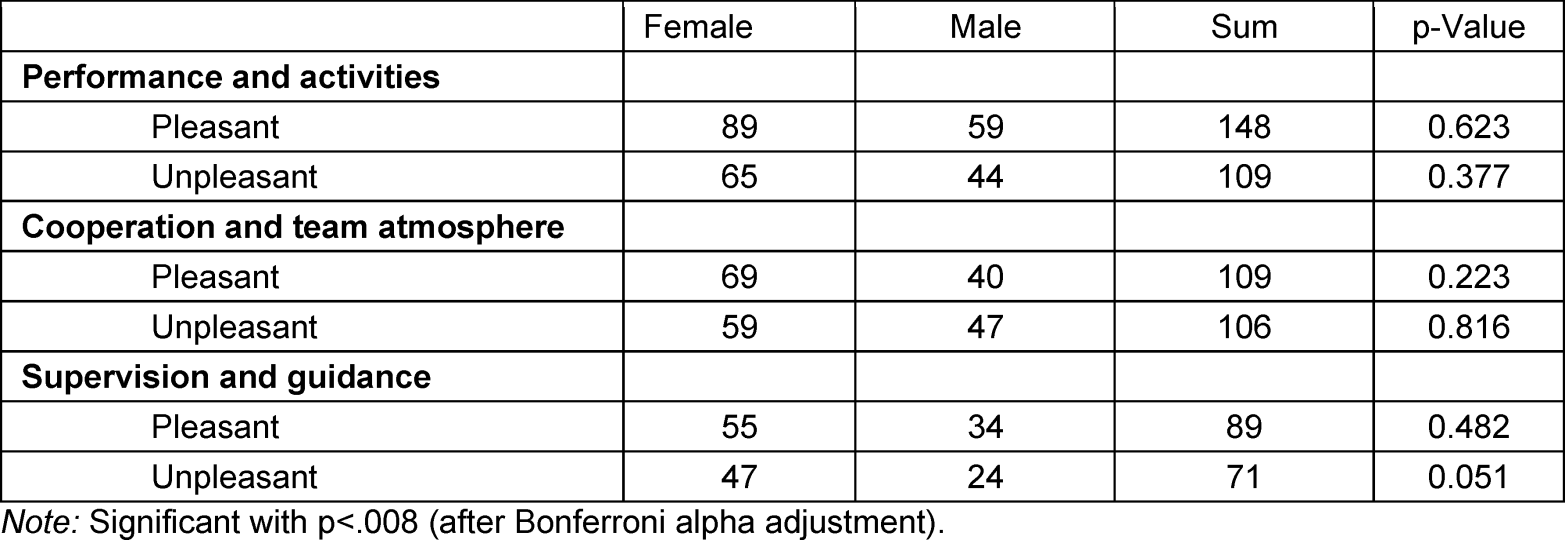
Gender differences (chi-squared test)

**Table 3:**
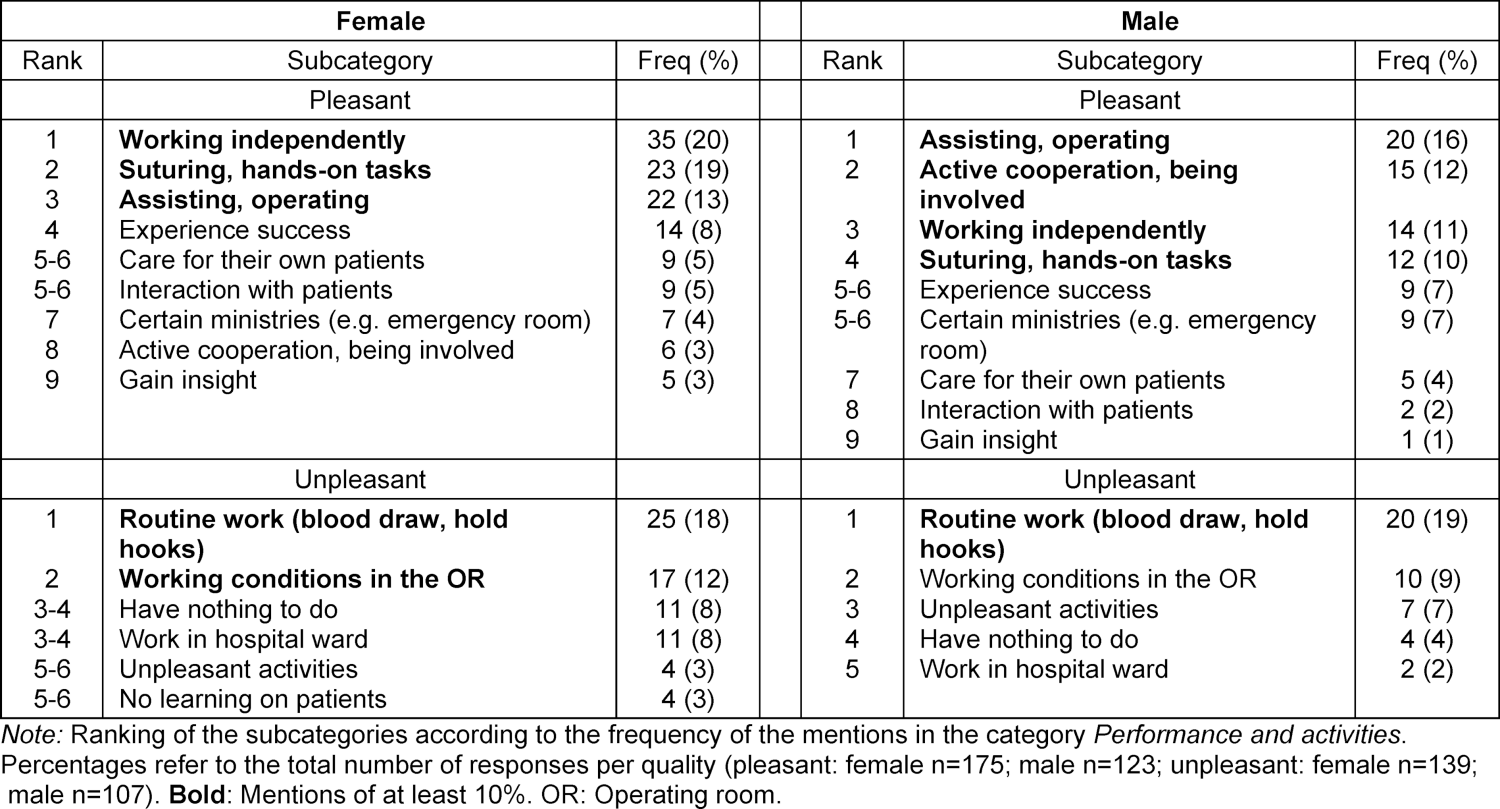
Performance and activities: sex/gender differences (quantitative content analysis)

**Table 4:**
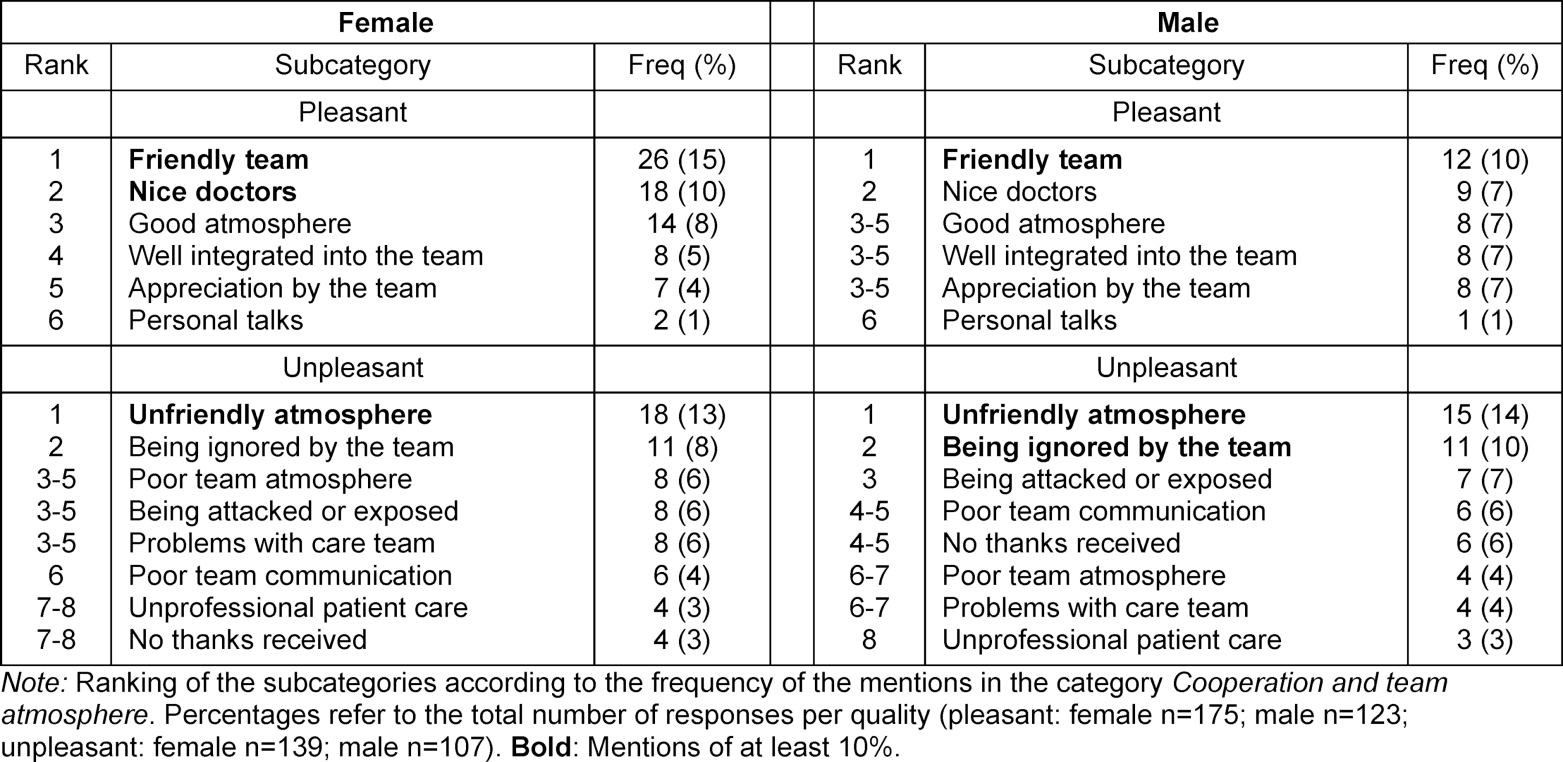
Cooperation and team atmosphere: sex/gender differences (quantitative content analysis)

**Table 5:**
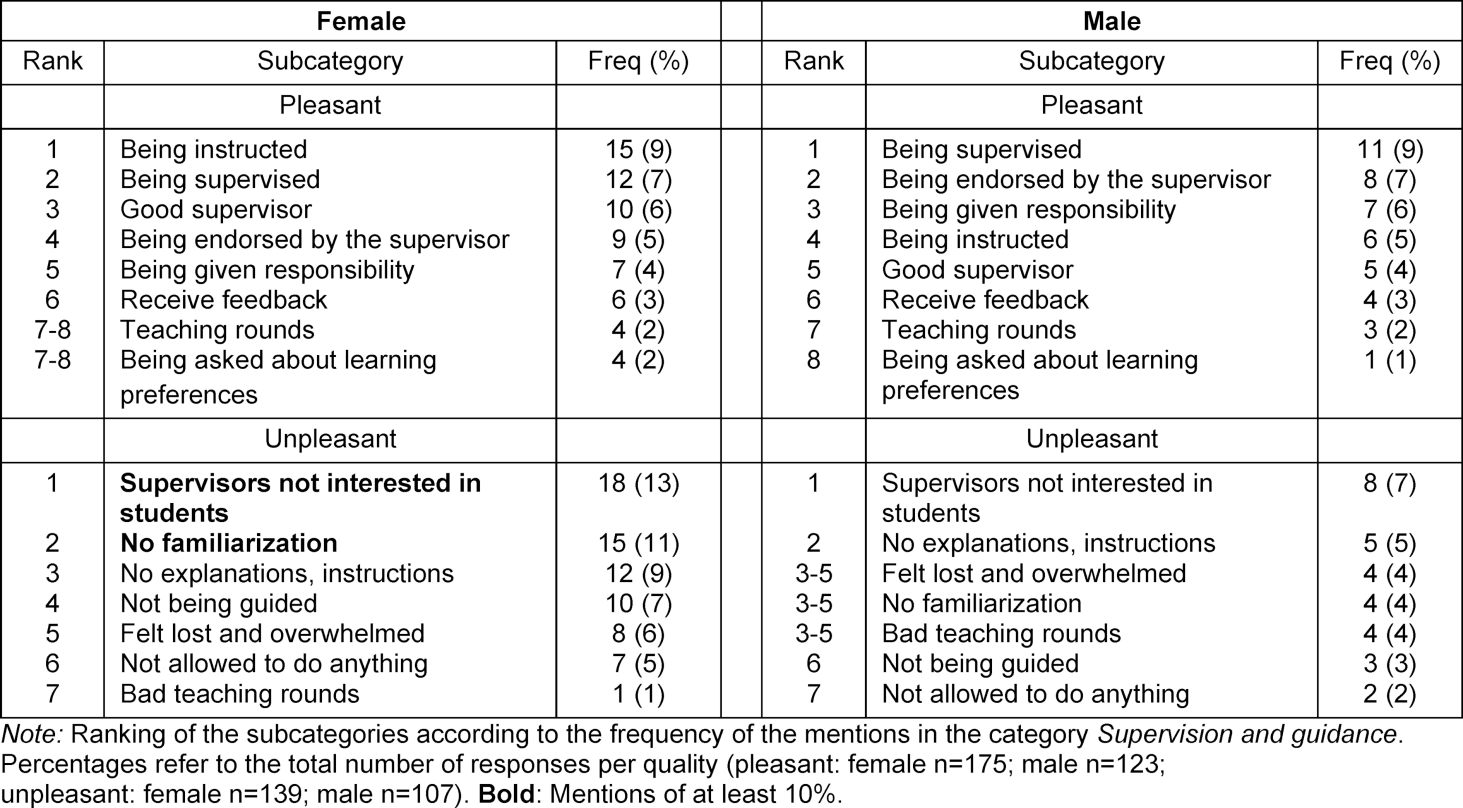
Supervision and guidance: sex/gender differences (quantitative content analysis)
